# A key role for the N/OFQ-NOP receptor system in modulating nicotine taking in a model of nicotine and alcohol co-administration

**DOI:** 10.1038/srep26594

**Published:** 2016-05-20

**Authors:** Andrea Cippitelli, Jennifer Schoch, Ginamarie Debevec, Gloria Brunori, Nurulain T. Zaveri, Lawrence Toll

**Affiliations:** 1 Torrey Pines Institute for Molecular Studies, 11350 SW Village Parkway, Port St. Lucie, FL 34987, USA; 2 Astraea Therapeutics, 320 Logue Avenue, Suite 142, Mountain View, CA 94043, USA

## Abstract

Alcohol and nicotine are often co-abused. Although the N/OFQ-NOP receptor system is considered a potential target for development of drug abuse pharmacotherapies, especially for alcoholism, little is known about the role of this system in nicotine dependence. Furthermore, the effect of prior history of nicotine dependence on subsequent nicotine and alcohol taking is understudied. Using an operant co-administration paradigm, in which rats concurrently self-administer nicotine and alcohol, we found that nicotine dependent rats increased nicotine self-administration over time as compared to non-dependent animals, while patterns of alcohol lever pressing did not change between groups. Pretreatment with the potent NOP receptor agonist AT-202 (0.3–3 mg/kg) increased nicotine lever pressing of both dependent and non-dependent groups, whereas the selective antagonist SB612111 (1–10 mg/kg) elicited a clear reduction of nicotine responses, in both dependent and non-dependent rats. In parallel, AT-202 only produced minor changes on alcohol responses and SB612111 reduced alcohol taking at a dose that also reduced locomotor behavior. Results indicate that a history of nicotine dependence affects subsequent nicotine- but not alcohol-maintained responding, and that NOP receptor antagonism, rather than agonism, blocks nicotine self-administration, which strongly suggests a critical role for the endogenous N/OFQ in the modulation of nicotine reinforcement processes.

Worldwide, 6 million people, including 480,000 in the United States, die premature deaths from smoking-induced disease each year. Data are expected to worsen to more than eight million by 2030. In addition to illness and mortality, tobacco addiction produces an impressive burden in terms of direct medical costs and lost productivity with direct medical costs averaging $170 billion/yr and lost productivity costs averaging $150 billion/yr by 2010^1^,^2^. One factor that influences this dramatic scenario is that, due to the very addictive nature of cigarettes, current FDA-approved pharmacotherapies to facilitate smoking cessation, including nicotine replacement therapy (NRT; nicotine patches, gums, etc.), the antidepressant bupropion, and the nicotinic acetylcholine receptor (nAChR) partial agonist varenicline (Chantix), have very low success rates when examined over a year time period, in the range of 10%, 15% and 20–25% for NRT, bupropion and varenicline respectively^3^,^4^. Clearly novel therapeutics that may have better efficacy or perhaps could synergize with current pharmacotherapies could be of clinical value. Furthermore, tobacco addiction is often accompanied by dependence to other abused drugs. Alcohol is the most common substance to be co-abused with tobacco^5^, which exponentially increases the burden.

Because of the high level of co-morbidity, it is reasonable to postulate that pharmacotherapies affecting nicotine abuse might also be effective for treatment of alcoholics. In fact that seems to be the case. The α4β2 nAChR partial agonist varenicline, which was originally developed as a smoking cessation medication^4^, appears to be equally efficacious for reducing alcohol use^6^,^7^. Likewise, the mu opiate antagonist naltrexone is approved for treatment of opioid and alcohol abuse, but in some clinical trials has shown effectiveness for reducing smoking as well^8^. Another receptor system that has been extensively explored with respect to drug abuse is the fourth member of the opioid receptor family, the NOP receptor, and its endogenous ligand nociceptin/orphanin FQ (N/OFQ)^9^,^10^,^11^. NOP receptors and N/OFQ are found in high concentrations in brain regions known to be involved in drug abuse, including the ventral tegmental area (VTA), nucleus accumbens (NAcc), prefrontal cortex, amygdala, and medial habenula (MHb)^12^,^13^,^14^,^15^. Furthermore, N/OFQ and small molecule agonists have been demonstrated to block conditioned place preference (CPP) of morphine, alcohol, cocaine, and methamphetamine^16^,^17^,^18^,^19^,^20^. This is consistent with the observation that NOP receptor agonists also block a drug-induced increase in extracellular dopamine in the rat NAcc^21^,^22^. Although the effect of a NOP receptor agonist on drug-induced CPP is well established, the ability of N/OFQ and NOP agonists to block drug self-administration is less clear. N/OFQ was ineffective in blocking heroin self-administration^23^, and its effect on alcohol self-administration is equivocal. In most studies NOP receptor agonists were effective in attenuating alcohol consumption only in genetic models of alcohol preferring rats, such as the Indiana P rat and the Marchigian Sardinian alcohol-preferring (msP) rat^24^,^25^ or in rats with a previous history of alcohol dependence but not in unselected or non-dependent rat lines^26^,^27^. This suggests that alcohol-induced or innate anomalies in the brain NOP-N/OFQ system could lead to increased alcohol consumption and to increased sensitivity to the effects of NOP receptor agonists. Surprisingly, although a role of endogenous nociceptin in modulating the acute effects of nicotine has been hypothesized based an increase in the sensitivity to nicotine in NOP receptor knockout mice^28^, it is unknown whether NOP receptor agonists are able to block nicotine reward. Nevertheless, NOP receptor agonists are often considered potential targets as drug abuse medications^29^.

In order to investigate co-morbidity of nicotine and alcohol, a co-self-administration model has been developed in which rats press one lever to deliver nicotine and the second lever to deliver alcohol^30^. Using a similar methodology we determined that the α3β4 nAChR partial agonist AT-1001 could attenuate nicotine self-administration, while it was mostly inactive in blocking alcohol self-administration when both drugs are available in the operant chamber. This is unlike varenicline, which was equally effective in blocking both nicotine and alcohol taking^31^. Additional co-administration studies were initiated to investigate two parameters of co-administration of nicotine and alcohol. First, it has never been established whether dependence upon one drug affects the use of the other. Second, we were interested in examining the effectiveness of NOP agonists in attenuating nicotine and alcohol self-administration in non-dependent rats, and those previously dependent upon nicotine, through chronic application of nicotine patches. The current results indicate that prior nicotine dependence affects nicotine but not alcohol self-administration, and surprisingly that NOP receptor antagonists, rather than agonists, can block nicotine self-administration.

## Results

### Nicotine dependence state

**Figure 1:**
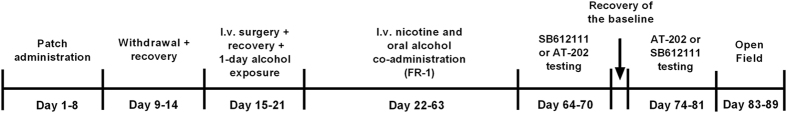
Timeline of the experiment. All animals initially underwent a chronic nicotine treatment by application of nicotine patches or control procedure. Occurrence of withdrawal was verified following nicotine discontinuation. All rats were subsequently implanted with an i.v. catheter and exposed to a 10% alcohol solution in their home cages prior to beginning operant i.v nicotine and oral alcohol co-self-administration (20 sessions in 6 weeks). Both the NOP receptor agonist AT-202 and the antagonist SB612111 were tested according to a Latin Square design. A test for locomotor activity was ultimately conducted to determine whether the observed effects of SB612111 were behaviorally specific.

Figure 1 shows the timeline of the experiment.

Changes in body weight are thought to be a hallmark of withdrawal intensity^32^. As evidence of successful nicotine dependence induction, weight gain during withdrawal, occurrence of somatic withdrawal signs and nicotine as well as cotinine blood levels were assessed in the patch exposed and control rats.

**Figure 2:**
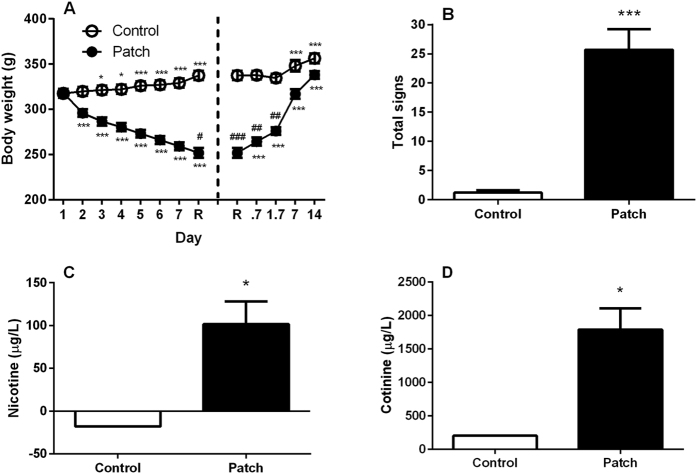
Dependence state upon nicotine following 7.3 days of chronic exposure to transdermal nicotine patches (5.2 mg/rat/day). Dependence was verified (**A**) by measuring body weight loss and gain across the intoxication period and upon nicotine discontinuation, respectively. Data are the mean (±SEM) grams (g) of body weight during intoxication from day 1 to the time of removal (R) and at 16 hours (0.7 days), 40 hours (1.7 days) 7 and 14 days following R. *p < 0.05, ***p < 0.001 difference from day 1 or R in both nicotine exposed (patch, N = 9) and non-exposed (control, N = 9). ^#^p < 0.05, ^##^p < 0.01, ^###^p < 0.001 difference from control. Dependence was also verified (**B**) by observing spontaneous withdrawal signs 16h after patch removal. Rats previously exposed to nicotine showed increased occurrence of overall withdrawal signs compared to non-exposed controls. Overall withdrawal signs were obtained for each animal by accumulating different categories of somatic withdrawal manifestations (cheek tremors, teeth chattering, head/body shakes, gasps and writhes). ***p < 0.001 difference from control. Finally, to control for effective nicotine release by patches (**C**) blood nicotine and (**D**) cotinine levels in the blood were verified two hours following the last change of patches. Nicotine exposed rats showed elevated blood nicotine and cotinine concentrations expressed as mean (±SEM) micrograms per liter (μg/L) compared to non-exposed rats. The signal detected in the control group for both nicotine and cotinine were not significantly lower or over the blank standard, respectively. *p < 0.05 difference from control. For detailed statistics, see “Results”.

The nicotine dependence induction following chronic application of nicotine patches produced gradual decrease in body weight followed by increased weight gain upon nicotine discontinuation. Overall ANOVA revealed an effect of “patch” [F_(1,16)_ = 51.3, p < 0.001] accompanied by effect of “day” [F_(7,112)_ = 127.3, p < 0.001] and interaction “patch x day” [F_(7,112)_ = 376.0, p < 0.001]. On post hoc analysis the dependence induction procedure caused a progressive drop in body weight from day 1 (318.2 ± 3.8 g) to the time of patch removal (251.8 ± 5.4 g, p < 0.001 for each day) whereas control animals maintained their normal weight gain pattern (from 317.6 ± 4.8 to 337.4 ± 5.0). At the time of removal difference in body weight was observed between the patch group as compared to the control group (p < 0.05). Following patch removal ANOVA revealed a significant effect of “removal” [F_(1,16)_ = 62.6, p < 0.001] and “time” [F_(4,64)_ = 342.7, p < 0.001], accompanied by interaction “removal x time” [F_(4,64)_ = 133.5, p < 0.001]. Post hoc analysis displayed an abrupt weight increase of the animals previously exposed to nicotine at all time points examined (0.7, 1.7, 7, 14 days after removal, p < 0.001). One week of nicotine discontinuation was sufficient to rapidly return body weight of animals with a history of nicotine exposure to the levels of nicotine naïve animals (Fig. 2A).

Another symptom of the dependence state is the spontaneous occurrence of somatic signs of withdrawal upon drug discontinuation. The patch procedure spontaneously produced a substantial increase of overall somatic withdrawal signs compared to controls, when measured 16 h after patch removal (p < 0.001, Fig. 2B). In particular, rats receiving nicotine patches differed significantly from control rats in cheeks tremors (p < 0.001), teeth chattering (p < 0.001), writhes (p < 0.01) and gasps (p < 0.01). The only dependent variable that seemed to be not affected by nicotine was head/body shakes (Table 1).

To control for effective nicotine release by patches, blood nicotine and cotinine levels were verified. Application of nicotine patches resulted in increased levels of nicotine and cotinine in the blood. Average nicotine concentration two hours following the last change of patches was 101.5 ± 26.5 μg/L. At the same time point, level of cotinine, the primary metabolite of nicotine, was as high as 1789.6 ± 317 μg/L suggesting accumulation of the metabolite in the blood throughout the chronic patch procedure. The non-parametric Mann–Whitney *U* test revealed significant difference between the patch and the control group both in blood nicotine (p < 0.05, Fig. 2C) and cotinine (p < 0.05, Fig. 2D) concentrations. The signal detected in the control group for both nicotine and cotinine were not significantly different than the blank standard.

### Operant co-administration of intravenous (i.v.) nicotine and oral alcohol

**Figure 3:**
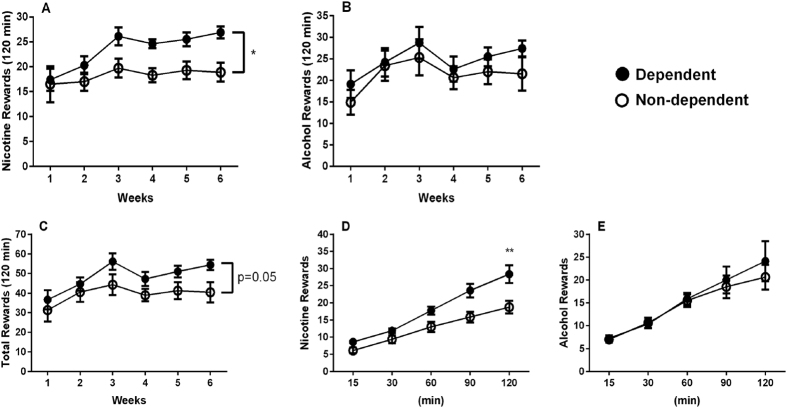
Operant co-administration of intravenous (i.v.) nicotine (30 μg/kg/inf.) and oral alcohol (10% v/v) in nicotine-dependent (N = 9) and non-dependent (N = 9) rats under a FR-1 schedule. Twenty 120-min sessions were conducted two to five times per week for 6 weeks. Data are reported as the mean (±SEM) number of (**A**) nicotine rewards, (**B**) alcohol rewards and (**C**) total rewards. Panels (**D**,**E**) show the time course of nicotine and alcohol rewards, respectively, earned within a representative 120-min session. *p ≤ 0.05, **p < 0.01 difference between groups. For detailed statistics, see “Results”.

Figures 3A,B show patterns of nicotine and alcohol reinforcements, respectively, over 6 weeks of nicotine and alcohol co-administration sessions in which animals, previously exposed (nicotine dependent) or not exposed (non-dependent) to nicotine patches, performed 20 sessions according to a randomized weekly exposure (two to five sessions per week). We performed separate repeated-measures ANOVAs for each drug. For nicotine-reinforced responding ANOVA indicated a significant main effect of “group” (nicotine dependent vs. non-dependent rats: F_(1,16)_ = 7.7, p < 0.05) and “week” (response across the 6 weeks: F_(5,80)_ = 5.5, p < 0.001). However, there was no interaction between the two factors (F_(5,80)_ = 1.5, NS). In contrast, alcohol-reinforced responding did not change between groups (F_(1,16)_ = 1.3, NS) and interaction “group x week” was not found (F_(5,80)_ = 0.2, NS), though there was effect of “week” (F_(5,80)_ = 3.4, p < 0.01). Analysis of total rewards earned showed a barely increased response (F_(1,16)_ = 4.2, p = 0.05), due to increased responses for only nicotine (Fig. 3C). These data indicate that rats with a history of nicotine dependence take more nicotine but not alcohol than non-dependent rats when both drugs are simultaneously available.

To better understand the rate at which animals self-administer nicotine and alcohol, we looked at the time course of responding across the 120-min sessions. Since responding for nicotine and alcohol were very consistent throughout sessions, particularly starting by the third week, we chose one representative session (session #17) and analyzed the hourly time course of nicotine infusions and alcohol rewards in nicotine dependent and non-dependent animals. ANOVA revealed a significant interaction “group” x “hour” (F_(1,15)_ = 8.1, p < 0.05) with post hoc comparisons showing that nicotine dependent rats pressed for nicotine more vigorously than non-dependent animals during the second hour of the session (p < 0.01), while responding during the initial 60 min was similar between groups. In contrast, alcohol-maintained responses did not differ between groups over time (F_(1,15)_ = 0.2, NS). A detailed time course of lever pressing for nicotine and alcohol is shown in Fig. 3D,E, respectively.

### Effect of the NOP receptor agonist AT-202 on nicotine and alcohol co-administration

**Figure 4:**
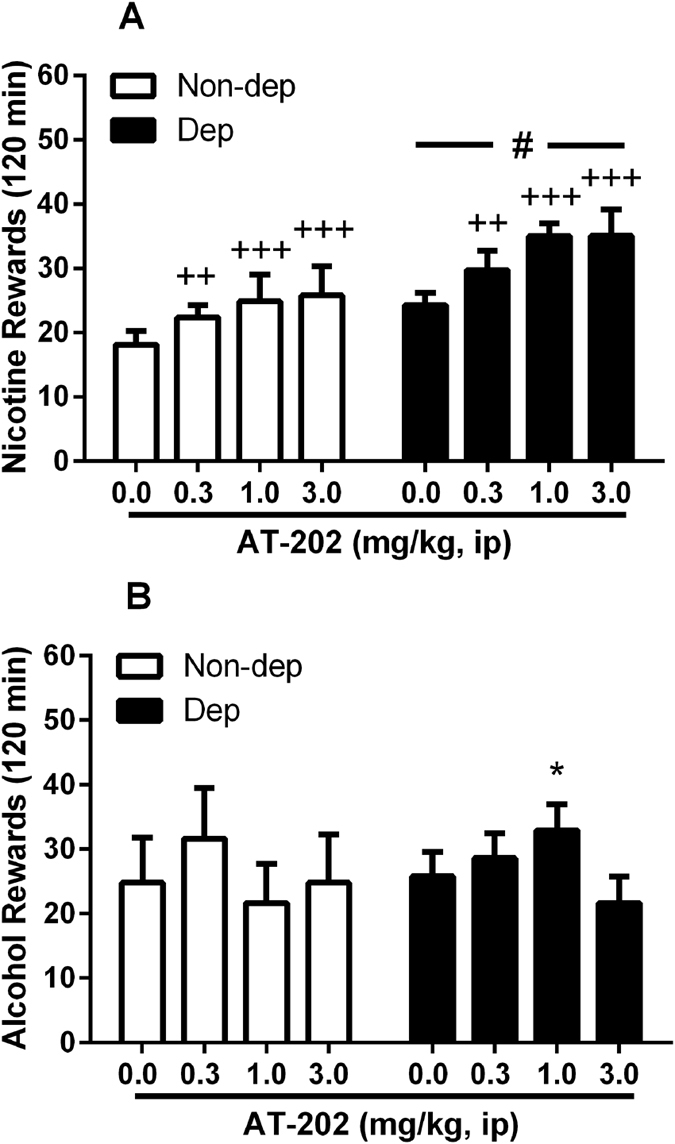
Operant co-administration of nicotine and alcohol and modulation by NOP agonist AT-202. (**A**) Values are mean (±SEM) of nicotine rewards (infusions) in 120 min following i.p. administration of AT-202. An increase in nicotine-reinforced lever pressing under a FR-1 schedule following pretreatment with 0.3–3 mg/kg AT-202 was observed in both nicotine-dependent (Dep, N = 9) and non-dependent (Non-dep, N = 9) rats where ^++^p < 0.01, ^+++^p < 0.001 indicate significant differences from vehicle (0.0 mg/kg) collapsed across “group” (main effect of “treatment”). Also, an increase in nicotine rewards was observed in dependent compared to non-dependent animals, where ^#^p = 0.05 indicates a difference from Non-dep group collapsed across “treatment” (main effect of “group”). (**B)** Values are mean (±SEM) of alcohol rewards. Alcohol self-administration was only slightly increased only in the nicotine-dependent group. Although the overall ANOVA revealed an interaction “treatment x group” effect, post hoc tests indicated a significant increase in responding following treatment with 1.0 mg/kg AT-202 in dependent animals compared to appropriate vehicle group (*p < 0.05). For detailed statistics, see “Results”.

Because NOP receptor agonists are effective at inhibiting the reinforcing and motivational effects of alcohol particularly in rats with a previous history of alcohol dependence^26^,^27^, AT-202 was tested on the co-administration paradigm in nicotine dependent and non-dependent animals to determine whether it was equally effective in blocking nicotine-taking behavior. Surprisingly, as shown in Fig. 4A, pretreatment with the NOP agonist increased rather than decreased nicotine-maintained lever pressing and did so in both nicotine dependent and non-dependent groups. Overall ANOVA revealed a main effect of “treatment” (various doses of AT-202: F_(3,48)_ = 12.4, p < 0.001) and a main effect of “group” (nicotine dependent vs. non-dependent: F_(1,16)_ = 4.5, p = 0.05), confirming that nicotine responses of nicotine dependent rats were higher than the non-dependent rats. There was no interaction between the two factors [i.e., the “group” factor did not influenced the effect of AT-202 treatment (F_(3,48)_ = 0.5, NS)]. On post hoc analysis conducted on the individual independent variable of “treatment”, all three doses examined elicited enhanced nicotine-taking behavior as compared to vehicle (p < 0.01 for 0.3 mg/kg and p < 0.001 for 1.0 and 3.0 mg/kg dose). This effect of AT-202 was also observed for the alcohol-associated lever [main effect of “treatment” (F_(3,48)_ = 5.0, p < 0.01)] where a significant interaction “treatment” x “group” was detected (F_(3,48)_ = 6.8, p < 0.001). However, post hoc pairwise comparisons only showed a weak increase in alcohol responding of nicotine dependent rats after pretreatment with AT-202 at a single 1.0 mg/kg dose (p < 0.05, Fig. 4B). These observations suggest that administration of NOP receptor agonist clearly and reliably enhances nicotine lever pressing whereas alcohol lever pressing is altered to a lesser extent and only in rats previously made dependent upon nicotine.

### Effect of the NOP receptor antagonist SB612111 on nicotine and alcohol co-administration

**Figure 5:**
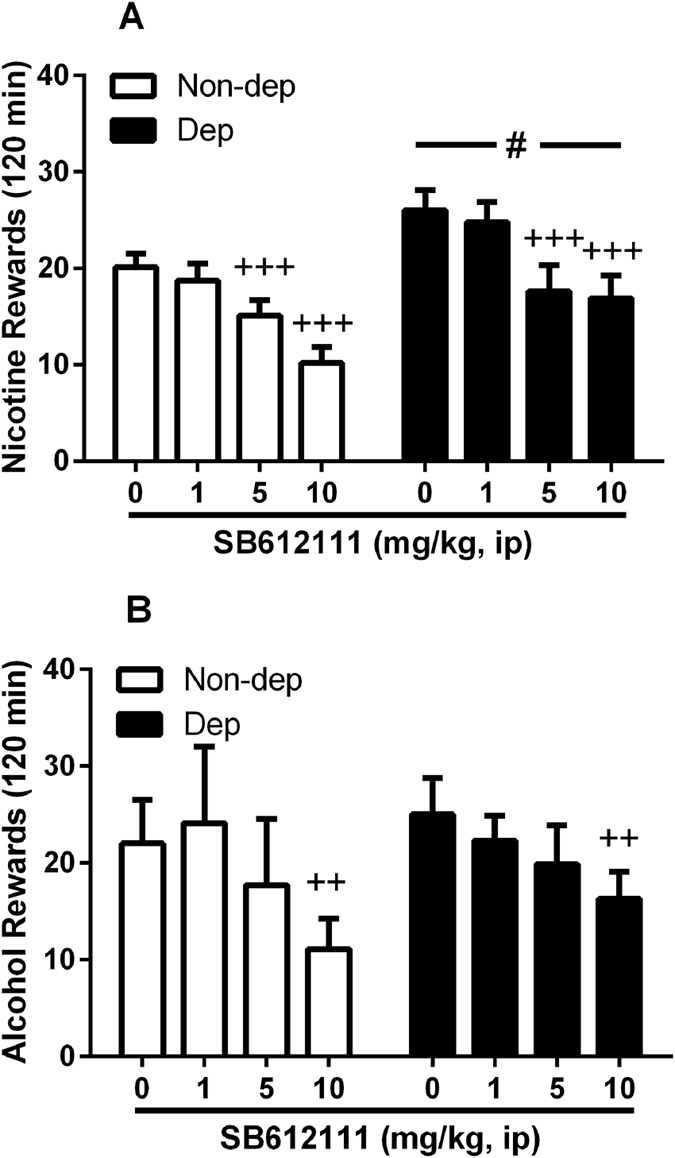
Operant co-administration of nicotine and alcohol and modulation by NOP antagonist SB612111. (**A)** Values are mean (±SEM) of nicotine rewards (infusions) in 120 min following i.p. administration of SB612111. A decrease in nicotine-reinforced lever pressing under a FR-1 schedule following pretreatment with 5–10 mg/kg NOP receptor antagonist was observed in both nicotine-dependent (Dep, N = 9) and non-dependent (Non-dep, N = 9) rats where ^+++^p < 0.001 indicates a significant difference from vehicle (0.0 mg/kg) collapsed across “group” (main effect of “treatment”). Also, an increase in nicotine rewards was observed in dependent compared to non-dependent animals, where ^#^p < 0.05 indicates a difference from Non-dep group collapsed across “treatment” (main effect of “group”). (**B)** Values are mean (±SEM) of alcohol rewards. The overall ANOVA revealed a main effect of treatment, where post hoc tests indicated that only 10 mg/kg SB612111 decreased alcohol reward compared to vehicle controls collapsed across “group” (^++^p < 0.01). For detailed statistics, see “Results”.

This experiment was carried out to test the effects of the manipulation of the endogenous N/OFQ signaling on nicotine and alcohol co-self-administration, by blocking the NOP receptor with the antagonist SB612111. Results clearly showed that SB612111 produced an opposite response from that seen for the NOP agonist AT-202, by eliciting a reduction of operant responding for nicotine in both dependent and non-dependent animals [main effect of “treatment”: (F_(3,48)_ = 22.8, p < 0.001); main effect of “group”: (F_(1,16)_ = 5.0, p < 0.05; once again, nicotine responses of nicotine dependent rats were higher than the non-dependent rats); no interaction (F_(3,48)_ = 1.0, NS), Fig. 5A]. On post hoc analysis of the individual independent variable of “treatment”, 5 and 10 mg/kg doses of SB612111 but not 1 mg/kg were effective in reducing nicotine taking (p < 0.001 for both). Concurrently, alcohol lever pressing was also reduced but to a lesser extent than nicotine. ANOVA showed main effect of “treatment” (F_(3,48)_ = 5.8, p < 0.01) accompanied by no effect of “group” (F_(1,16)_ = 0.1, NS) or interaction “treatment” x “group” (F_(3,48)_ = 0.6, NS). Post hoc analysis of the individual “treatment” factor displayed effectiveness of SB612111 at 10 mg/kg (p < 0.01) but not following administration of 1 and 5 mg/kg doses (Fig. 5B). These results indicate that the pharmacological manipulation of NOP receptors with agonists or antagonists primarily modulates nicotine-taking behavior rather than alcohol taking, an effect, which is common to both rats previously dependent upon nicotine and non-dependent. Current data also suggest that antagonist activity rather that the agonist activity at NOP receptor reduces nicotine self-administration.

### Locomotor effects of SB612111

To determine whether SB612111 co-administration data were behaviorally specific, locomotor effects of SB612111 were examined in the open field paradigm. Under familiarity conditions, rats treated with the NOP receptor antagonist prior to allowing exploration of an open arena showed a slightly, though statistically significant, decrease in the total distance travelled across the 10 min test (F_(2,16)_ = 6.7, p < 0.01, Table 2). Post hoc comparisons showed effect of the dose of 10 mg/kg (p < 0.01) but not 5 mg/kg dose. However, changes in distance travelled were not accompanied by changes of other variables such as time of immobility (F_(2,16)_ = 3.1, NS). Thus, the observed reduction of both nicotine and alcohol self-administration following SB612111 treatment at the dose of 10 mg/kg could be influenced by a weak reduction of the rat locomotor behavior.

## Discussion

In light of the very high incidence of co-morbidity of alcohol and nicotine, we developed a two lever model of alcohol and nicotine co-administration in order to investigate the influence of one drug on administration of the other and to examine the effect of NOP receptor activation or inhibition on self-administration of both abused drugs. Previous experiments by Le and colleagues, using this model, had demonstrated that the order of drug initiation independently affected the self-administration of alcohol and nicotine^33^. Because it has been demonstrated that a prior dependence upon alcohol leads to an increase in alcohol self-administration^34^,^35^, and because of high co-morbidity, we used this model to explore the effect of prior dependence upon nicotine on both nicotine and alcohol self-administration (see Fig. 1 for a Timeline for drug administration). The method we used to induce nicotine dependence was a commercially available nicotine patch, as we have described previously^36^, rather than the more common Alzet mini-pump^37^,^38^. Although the patch may not regulate nicotine delivery with the precision of a mini-pump, it is much more relevant to the human condition, and as seen in Fig. 2, produces reliable and consistent withdrawal upon removal of the patch.

The results of the self-administration experiments demonstrated that, over the course of three to six weeks, nicotine self-administration was increased in the nicotine post-dependent compared to the non-dependent group, while alcohol lever pressing did not change. These results demonstrate that, consistent with mechanisms underlying alcohol dependence^39^, chronic nicotine treatment leading to dependence, may produce neuroadaptation that, in turn, induces increased nicotine self-administration in the post-dependent state. Escalation of nicotine self-administration has been previously shown in animals given intermittent periods of abstinence with extended access to nicotine^40^. Short (2-hour) access, described here, did not produce a similar escalation. However, by the third week the group with a prior history of nicotine had a stably increased self-administration, compared to the non-dependent group, following our intermittent regimen of drug exposure. There are several possibilities that could explain this phenomenon. The increased nicotine use might be motivated by relief of a negative somatic and/or emotional state^41^, even after dependence is alleviated (after withdrawal symptoms have subsided), whereas a simultaneous increase in alcohol self-administration seems not to be required to provide relief. Alternatively, it is possible that a history of chronic nicotine leading to nicotine dependence, reduces the rewarding value of subsequent nicotine administration or elevates the hedonic set point, as described for cocaine^42^, thereby encouraging the subjects to administer greater amounts to reach an equivalent reward threshold. A third possibility that may account for the increased nicotine lever pressing in post-dependent rats is pharmacological tolerance (induced by chronic nicotine) to the aversive nature of high nicotine doses that translates into administration of greater amounts of nicotine in each session. It is known that the aversive nature of nicotine can be dependent upon the nAChR subunit composition. In mice in which the α5 subunit has been genetically eliminated, nicotine self-administration is increased at higher doses, suggesting a reduction in the aversive, but not rewarding aspect of nicotine administration^43^. Similar results are found with chemical ablation of the MHb, a brain region probably important for nicotine- but not alcohol-taking behavior^43^,^44^. Chronic nicotine-induced changes in the α5, or perhaps some other nAChR subunit could represent a mechanism by which nicotine dependence induces an increase in nicotine but not alcohol self-administration.

Activation of the NOP receptor, the fourth member of the opioid receptor family, has been demonstrated repeatedly to inhibit a drug-induced increase of dopamine in the NAcc^21^,^22^,^45^. Furthermore, both i.c.v. administered N/OFQ and systemically administered small molecule agonists block CPP induced by morphine, cocaine, methamphetamine and alcohol^16^,^18^,^19^,^24^,^46^,^47^,^48^. However, NOP receptor agonists have been less successful in blocking drug self-administration. For example, i.c.v. administered N/OFQ was ineffective in blocking heroin self-administration in rats^23^. Although it has been demonstrated several times that NOP receptor activation can block alcohol self-administration, experiments in all but one report^49^ were conducted in a genetic model of alcohol preferring rat, the msP rat, in which both the NOP receptor and CRF receptors are significantly altered^24^,^50^, or else in alcohol-dependent animals^27^. Consistent with the present findings, NOP receptor agonist did not modify alcohol self-administration in normal non-dependent Wistar rats^26^,^27^. To further investigate the effect of NOP receptor agonism on both nicotine and alcohol self-administration, post-dependent and non-dependent rats were pretreated with the potent NOP receptor agonist AT-202^16^,^51^ prior to self-administration sessions. Rather than attenuating self-administration of either nicotine or alcohol, AT-202 dose dependently increased responding for nicotine in both groups of animals, while significantly stimulating alcohol self-administration at only a single dose in the nicotine post-dependent group. Conversely, when the rats were pretreated with the high affinity and selective NOP receptor antagonist, SB612111^52^, nicotine self-administration was reduced in both post-dependent and non-dependent animals at 5 and 10 mg/kg and alcohol self-administration was reduced when both groups of rats were treated with 10 mg/kg SB612111, a dose also found to reduce, though weakly, locomotor behavior.

The mechanism by which the NOP receptor antagonist reduces nicotine and alcohol self-administration is unclear. NOP receptor agonists block cocaine- and morphine-induced increases in NAcc dopamine^21^,^22^ so one would assume that a similar result could be found for nicotine and alcohol, though this has apparently never been examined. In theory this should induce a reduction in the alcohol or nicotine reward during self-administration, as it apparently leads to a reduction in reward during the CPP experiment. On the other hand, there are significant differences in the nature of CPP and self-administration paradigms. First, most CPP experiments have demonstrated that NOP receptor agonists block the acquisition of CPP. In fact, Shoblock *et al*. demonstrated that the NOP receptor agonist Ro 64-6198 blocked the acquisition and reinstatement, but not the expression of morphine CPP^48^. In contrast, in the experiments described here and most self-administration studies, NOP receptor ligands are tested on the maintenance or expression of drug responding. So it is possible that NOP agonists block acquisition, but antagonists can block expression of drug taking or drug reward. Second, there are differences in the pattern of drug administration in the two models. CPP generates a rather modest history of drug administration (generally 3–4 days of administration), while self-administration requires many days or weeks of repeated administration prior to drug testing. Finally, CPP entails response-independent forced drug exposure, while self-administration uses volitional and self-regulated drug intake. It has been well documented that VTA dopamine cell excitability is enhanced to a greater degree by chronic self-administered vs. response-independent forced nicotine exposure^53^,^54^. Therefore, it is possible that NOP receptor agonists can adequately attenuate NAcc dopamine levels sufficient to block CPP but not self-administration of an abused drug.

**Figure 6:**
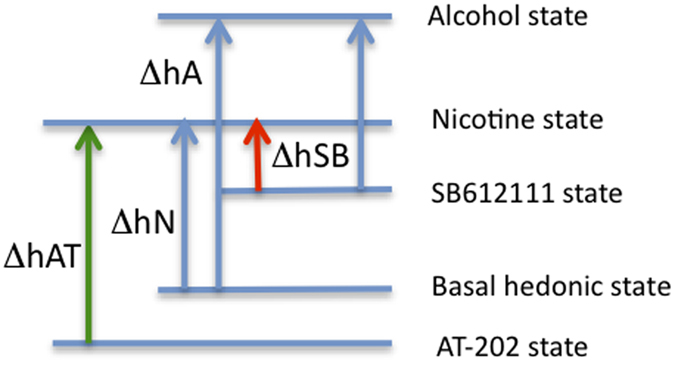
Model to explain NOP receptor agonist-induced stimulation of nicotine self-administration and antagonist-induced inhibition.

Our working hypothesis is that administration of NOP receptor agonists lower (presumably) basal dopamine levels and therefore reduce the basal hedonic state, while antagonists block endogenous N/OFQ, thereby increasing dopamine levels and increasing the hedonic state (see Fig. 6). We believe that nicotine is not a powerful reinforcer, which is consistent with the greater difficulty in inducing nicotine CPP^55^, and when the hedonic state is raised by treatment with SB612111, the amount of nicotine required to reach a nicotine reward threshold is reduced. By analogy, to reach the nicotine reward threshold, nicotine self-administration would be increased by prior AT-202 treatment. In other words, it is the magnitude of the drug-induced change in hedonic state (Δh) that regulates the extent to which an animal will work for drug, and therefore the level of expression of drug self-administration. Under this scenario, with a more rewarding drug, such as perhaps cocaine or alcohol, moderate changes in dopamine levels induced by pretreatment with NOP receptor agonists or antagonists would have a much reduced percentage effect on Δh and therefore drug self-administration, consistent with our alcohol data. Future microdialysis studies are planned to examine this hypothesis. Importantly, these data clearly demonstrate the that the two most employed “drug abuse” paradigms measure different responses based upon different behavioral parameters, and it is not clear which, if either, better models human drug-taking behavior.

In conclusion, we have demonstrated that a prior history of nicotine dependence increases subsequent nicotine but not alcohol self-administration in a dual nicotine/alcohol self-administration paradigm. Surprisingly, a NOP receptor agonist increased nicotine-taking behavior while an antagonist attenuated both nicotine- and alcohol-maintained responses. Although these experiments were not conducted in the individual systems, the effects of NOP receptor ligands appeared more potent and effective in altering nicotine rather than alcohol responses when both reinforcers were available. Additionally, the effects of the NOP receptor agonist as well as the antagonist were not different in nicotine post-dependent and non-dependent subjects suggesting that neuroadaptations underlying the increased nicotine taking of the post-dependent group implicated changes in nicotinic acetylcholine receptors rather than NOP receptors. Altogether, these results suggest that NOP receptor antagonists, rather than agonists, might have value as smoking cessation medications and potentially as treatment of alcohol use disorder.

## Methods

### Animals

Male Sprague-Dawley rats (N = 18, 200–225 g at their arrival) were obtained from Charles River (Portage, MI). Rats were housed two per cage in a room with a reverse 12-hour light/12-hour dark cycle (lights off at 7:30 AM). All experiments were conducted during the dark phase of the cycle. Animals were acclimated for seven days with water and chow (Teklad Diets, Madison, WI) provided *ad libitum* and handled for three days before the experiments were conducted. All animal experiments were carried out in accordance with the National Institutes of Health Guide for the Care and Use of Laboratory Animals. Consistent with these guidelines, ongoing statistical testing of data collected was used to minimize the number of animals used, within the constraints of necessary statistical power. All methods used were pre-approved by the Institutional Animal Care and Use Committee at the Torrey Pines Institute for Molecular Studies (Port Saint Lucie, FL).

### Drugs

SB612111 and AT-202 were synthesized and provided by Astraea Therapeutics (Mountain View, CA) and suspended in a vehicle containing 2% DMSO, and 98% hydroxypropyl cellulose (0.5% in distilled water). These compounds were administered in a 1 ml/kg volume injection and given by intraperitoneal (i.p.) route of administration. (−)-Nicotine hydrogen tartrate salt and alcohol were purchased from Sigma (St. Louis, MO). Alcohol was diluted in water and made available orally. Solution of nicotine for i.v. injection was obtained by dissolving (−)-Nicotine hydrogen tartrate in 0.9% saline and the pH adjusted to 7.0–7.4 with 5 M sodium hydroxide. Nicotine self-administration dose is reported as free base concentration. Nicotine patches (Equate, Step 1, 21 mg/day, WalMart Stores, Inc) were used for dependence induction.

### Dependence induction procedure

Three weeks after arrival, all rats were thoroughly shaved on the back, depilated with a depilatory lotion and cleansed with water as previously described^36^. Patches were divided into 4 equal parts so that 5.2 mg/rat/day of nicotine was administered by patch applied to the shaved region of rats (N = 9). Comparable doses were previously shown to produce sufficiently high blood nicotine and cotinine levels^56^ to elicit occurrence of reliable nicotine abstinence symptoms. Pieces of flexible fabric Band-Aid and waterproof tape were used to wrap the nicotine patch to improve its adherence to the rat’s back. Control rats (N = 9) were shaved and depilated, but only the Band-Aid and waterproof tape were placed on their backs. This application procedure was repeated for 7 consecutive days. On day 8, transdermal patches were applied for additional 8 hours and then removed to study nicotine withdrawal. Therefore, total duration of the procedure was 7.3 days.

### Weight gain during withdrawal

All animals were weighed each day when transdermal patches were replaced until the end of the exposure (removal). Recovery of body weight was monitored at 16 hours (0.7 days), 40 hours (1.4 days), 7 and 14 days following patch removal.

### Assessment of somatic withdrawal signs

Physical abstinence signs were determined 16 hours after the removal of nicotine patches^38^,^57^. The assessment of somatic signs was performed under a dim source of light by blind observation across 12 min. One observer counted the frequency of signs on a standard checklist of nicotine abstinence signs, as previously described^36^,^58^. The most frequently observed signs included cheek tremors, teeth-chattering, head/body shakes, writhing and gasping. Cheek tremors were counted no more frequently than once per ten seconds. Data are reported as average (±SEM) of number of episodes counted in 12 min.

### Nicotine and cotinine blood level assessment

To determine levels of nicotine and cotinine blood (100 μl) was collected from the tail vein two hours following the last change of patches (day 8) of the nicotine dependence procedure (further details in Supplementary Information).

### I.v. catheter implantation

One week after the end of the nicotine dependence induction procedure, all rats were subjected to i.v. catheterization. Under isoflurane anesthesia (Vetamac, Inc., Rossville, IN) an incision was made to expose the right jugular vein and a catheter made from silicon tubing (I.D. = 0.020 inches, O.D. = 0.037 inches) was subcutaneously positioned. After insertion into the vein, the proximal end of the catheter was anchored to the muscles underlying the vein with surgical silk. The distal end of the catheter was attached to a threaded cannula guide bent at a 90° angle that protruded from the rat’s back. The cannula was capped with plastic tubing and covered with threaded lightweight aluminum hood^59^. To maintain patency for the duration of the experiment, catheters were flushed daily with 0.2 mL of heparin (1000 UPS U/ml)-containing saline solution, which also contained enrofloxacin (0.7 mg/ml). Self-administration experiments began 1 week after surgery.

### Apparatus

Self-administration experiments were conducted in operant conditioning chambers (Med Associates, Inc., St. Albans, VT) enclosed in lit, sound attenuating, ventilated environmental cubicles (further details in Supplementary Information).

### Co-administration

Upon recovery from surgery rats were introduced to operant chambers and allowed to concurrently lever press for nicotine and alcohol on a fixed ratio 1 (FR1) schedule of reinforcement. During this 7-day recovery period to facilitate the acquisition of alcohol self-administration, rats were provided free-choice access to alcohol (10% v/v) and water for 1 day in their home cages to habituate them to the taste of alcohol^60^. Operant conditions used in this experiment were chosen from previous work^30^,^31^. Following each nicotine infusion (30 μg/kg/0.1 ml) a 20 s time-out (TO) period occurred, during which responses at the lever that delivered nicotine (right lever) did not lead to programmed consequences. Nicotine reinforcements were accompanied by concurrent illumination of a cue light to signal delivery of nicotine. Oral alcohol reinforcements (10% v/v, 0.1 ml) were accompanied by a flashing house light (1.0 s on-1.0 s off) with a TO period of 20 s during which responses at the lever that delivered alcohol (left lever) did not lead to programmed consequences. An intermittent tone (7 kHz, 70 dB) was sounded throughout the 60 min session. These 2-hour self-administration sessions were conducted two to five times per week for 6 weeks. This intermittent regimen has led other authors to demonstrate that chronic nicotine facilitates compulsive alcohol drinking in rats^60^. Consistent with this regimen of drug exposure data are reported as weekly average ± SEM number of nicotine and alcohol rewards in 120 min.

### Drug testing

After 20 co-administration sessions the NOP agonist AT-202 (0.0, 0.3, 1.0 and 3.0 mg/kg) or the antagonist SB612111 (0, 1, 5 and 10 mg/kg) were tested to study their effects in nicotine and alcohol co-administration. First, nine (5 nicotine-dependent and 4 non-dependent) rats were i.p. administered with AT-202 10 min before sessions using a within-subject Latin square design. Test sessions were conducted every other day to allow drug wash out. Then, the same rats were tested with SB612111 given i.p. 10 min before sessions according to an identical design. Among the AT-202 and the SB612111 testing one session was conducted to recovery the baseline of responding. In parallel, the other nine (4 nicotine-dependent and 5 non-dependent) rats were tested first with SB612111 and then with AT-202 according to an identical design.

### Locomotor activity: effect of SB612111

Upon completion of drug testing 9 rats were chosen from the group of dependents and non-dependents and used for assessment of locomotor activity in the open field. The open field apparatus consisted of a square box with an open top, painted black, 50 cm wide × 30 cm tall. The arena was dimly illuminated. The test consisted of an initial trial in which rats were allowed to explore the open arena for 10 min. Other three 10-min trials were conducted 30 min following injection of SB612111 (0, 5, 10 mg/kg). Doses of SB612111 were i.p. administered in a counterbalanced order (Latin square design) every other day. Each trial was recorded by a video camera suspended above the field and interfaced with a computerized tracking system using Ethovision®XT version 5 software (Noldus Information Technology, Wageningen, The Netherlands). For each trial, the total distance traveled and immobility time were measured.

### Data analysis

Body weight analysis during dependence induction and withdrawal were performed by two-way ANOVA where the within-subject factors were “day” or “time” and the between-subject variables were “patch” or “removal”. Occurrence of the somatic signs of nicotine withdrawal was analyzed by the non-parametric Mann–Whitney *U* test as well as blood nicotine and cotinine concentrations. On operant co-administration experiments analysis of nicotine and alcohol rewards were conducted separately. Number of nicotine, alcohol, total rewards and the time course of lever pressing within a representative co-administration session was analyzed by a two-way ANOVA that used “week” or “hour” as a within-subject factor and “group” (dependent vs. non-dependent) as between-subject factor. Similarly, two-way ANOVAs were used to analyze effects of AT-202 and SB612111 (within-subject factor) on nicotine and alcohol co-self-administration in dependent vs non-dependent rats (between-subject factor). Finally, locomotor activity variables were analyzed by one-way ANOVA that used “treatment” as the within-subject factor. The level of significance was always set at p < 0.05. ANOVAs were followed, where appropriate, by Student Newman-Keuls post hoc tests.

## Additional Information

**How to cite this article**: Cippitelli, A. *et al*. A key role for the N/OFQ-NOP receptor system in modulating nicotine taking in a model of nicotine and alcohol co-administration. *Sci. Rep.* **6**, 26594; doi: 10.1038/srep26594 (2016).

## Supplementary Material

Supplementary Information

## Figures and Tables

**Table 1:** Spontaneous nicotine withdrawal symptoms measured 16 hours after the chronic (7.3 days) exposure to transdermal nicotine patches (5.2 mg/day).

	Cheek tremors	Teeth chattering	Body/head shakes	Writhes	Gasps
Control	0.0 ± 0.0	0.0 ± 0.0	0.3 ± 0.2	0.0 ± 0.0	0.9 ± 0.4
Patch	7.6 ± 1.3***	7.6 ± 1.9***	1.4 ± 0.6	3.2 ± 1.1**	5.9 ± 1.3**

Signs were detected under a dim source of light by blind observation across 12 min. The most evident somatic signs included cheek tremors, teeth chattering, body/head shakes, writhing and gasping. Data are the mean (±SEM) episodes in 12 min. **p < 0.01, ***p < 0.001 difference from control group. For detailed statistics, see “Results”.

**Table 2:** Locomotor activity as measured in the open field test over a 10 min period following treatment with SB612111 (0, 5 and 10 mg/kg, i.p.).

	SB612111 0 mg/kg	SB612111 5 mg/kg	SB612111 10 mg/kg
Distance travelled (cm)	2519.8 ± 145.1	2446.6 ± 147.3	2189.4 ± 153.2**
Immobility (s)	244.7 ± 11.6	247.6 ± 11.9	269.7 ± 12.4

Doses of the NOP receptor antagonist were administered in a counterbalanced order (Latin square design) in animals become familiar to the open arena.

Significant changes were observed for total distance travelled (cm) but not for time of immobility (s). **p < 0.01 difference from vehicle (SB612111 0 mg/kg) group. For detailed statistics, see “Results”.
