# Supplementary material for: A key role for the N/OFQ-NOP receptor system in modulating nicotine taking in a model of nicotine and alcohol co-administration
Source: Sci Rep. 2016 May 20;6:26594. doi: 10.1038/srep26594 (PMC4873733; doi:10.1038/srep26594)
Supplement: Supplementary Information [file srep26594-s1.pdf]

Supplementary Information

"A key role for the N/OFQ-NOP receptor system in modulating nicotine taking in a model of nicotine and alcohol co-administration"

Andrea Cippitelli, Jennifer Schoch, Ginamarie Debevec, Gloria Brunori, Nurulain T. Zaveri, Lawrence Toll

Further Details of "Nicotine and cotinine blood level assessment"

Blood from 5 rats exposed to patches and 4 control rats was added into a corresponding microcentrifuge tube that contained 1.0 ml of cold acetonitrile with 50 ng/ml internal standard. Samples were centrifuged at 3,000 rpm for 5 minutes. The supernatant was dried in a speed vacuum. Samples were reconstituted in 100 µl of 5% acetonitrile in water. Next, samples were centrifuged at 13,000 rpm for 5 minutes. A volume of 10 µl supernatant was used for analysis. Analysis was conducted by using High Performance Liquid Chromatography (HPLC, 20AD Shimadzu Prominence)/ Tandem Mass Spectrophotometry (MS/MS, AbSciex 3200 QTrap). In brief, to achieve separation on the HPLC of cotinine, nicotine, and the internal standard reverse phase mode with a gradient of 5-50% acetonitrile over 10 minutes was used. Mobile phase A was 5mM ammonium acetate with 0.1% formic acid. Mobile phase B was acetonitrile with 0.1% formic acid. The MS/MS analysis was performed in Multiple Reaction Monitoring (MRM) mode using the two largest fragments of the parent compounds. The ratio of the analyte peak area to the internal standard peak area was calculated. Then, a linear standard curve was made by plotting known concentrations versus peak area ratio. Each individual rat's nicotine and cotinine concentrations were calculated accordingly. The final values were converted to µg/L.

Further Details of "Apparatus"

Each chamber was equipped with two retractable levers located in the front panel, laterally to a drinking reservoir. Chambers were also equipped with auditory stimuli presented via a speaker

and visual stimuli located above the levers (cue lights) and near the top of the chamber opposite the lever on the front panel (house light). Infusions occurred by means of syringe pumps (Med Associates, Inc., St. Albans, VT) and liquid swivels (Instech Solomon, Plymouth Meeting, PA), connected to plastic tubing protected by a flexible metal sheath for attachment to the external catheter terminus. During co-administration of intravenous nicotine and oral alcohol the chambers were equipped with two active levers and two infusions pumps, one that delivered i.v. nicotine (0.1 ml) and one that delivered alcohol (0.1 ml) into the drinking receptacle. Thus, appropriate responding on the right lever resulted in activation of the pump containing nicotine while responding on the left lever resulted in activation of the pump that released alcohol, which was connected to the drinking receptacle through a PE-160 tube. A microcomputer controlled the delivery of reinforcers, presentation of auditory and visual stimuli, and recording of the behavioral data.
